# Erratum: Caroline Good; et al.; A Cultural Conscience for Conservation. *Animals* 2017, 7, 52

**DOI:** 10.3390/ani7080058

**Published:** 2017-08-04

**Authors:** Caroline Good, Dawn Burnham, David W. Macdonald

**Affiliations:** Wildlife Conservation Research Unit, University of Oxford, Oxford OX13 5QL, UK; dawn.burnham@zoo.ox.ac.uk (D.B.); david.macdonald@zoo.ox.ac.uk (D.W.M.)

The authors wish to make the following change to their paper [1]. The percentage numbers reflected the quantity in the original version. The correct percentage is displayed in the following:

**Figure 2:**
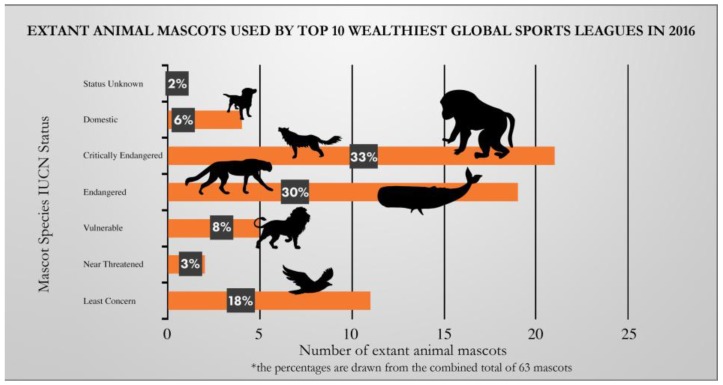
The extant animal mascots of the world′s top 10 wealthiest sports leagues.

The bars depicted in Figure 2 are the correct lengths. The change does not alter the point or message of the figure. We apologize for any inconvenience caused to the readers by this error. The manuscript will be updated and the original will remain online on the article webpage.
